# 2-Cyclo­pentyl­idenehydrazine­carboxamide

**DOI:** 10.1107/S1600536812034599

**Published:** 2012-08-11

**Authors:** Hoong-Kun Fun, Wan-Sin Loh, Mahesh Padaki, Arun M. Isloor, Nishitha A. Isloor

**Affiliations:** a X-ray Crystallography Unit, School of Physics, Universiti Sains Malaysia, 11800 USM, Penang, Malaysia; b Medicinal Chemistry Laboratory, Department of Chemistry, National Institute of Technology Karnataka, Surathkal, Mangalore 575 025, India; c Biotechnology Division, Department of Chemical Engineering, National Institute of Technology Karnataka, Surathkal, Mangalore 575 025, India

## Abstract

The asymmetric unit of the title compound, C_6_H_11_N_3_O, consists of two independent mol­ecules in which the cyclo­pentane rings adopt envelope conformations with CH_2_ grouping as the flap and the semicarbazone groups are essentially planar, with maximums deviation of 0.0311 (12) and 0.0285 (12) Å. In the crystal, N—H⋯O, N—H⋯N and C—H⋯O hydrogen bonds link the mol­ecules to form sheets lying parallel to the *ab* plane.

## Related literature

For background to the biological activity of semicarbazones, see: Dogan *et al.* (1999 ▶); Pandeya & Dimmock (1993 ▶); Pandeya *et al.* (1998 ▶); Yogeeswari *et al.* (2004 ▶); Sriram *et al.* (2004 ▶); Fun *et al.* (2011 ▶). For related structures, see: Fun *et al.* (2009*a* ▶,*b* ▶). For further synthetic details, see: Furniss *et al.* (1978 ▶). For the stability of the temperature controller used in the data collection, see: Cosier & Glazer (1986 ▶).
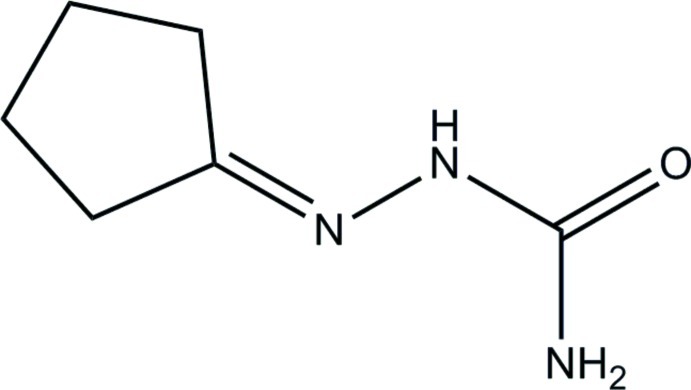


## Experimental

### 

#### Crystal data

C_6_H_11_N_3_O
*M* *_r_* = 141.18
Monoclinic, 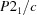

*a* = 8.9507 (1) Å
*b* = 10.7929 (2) Å
*c* = 15.0204 (2) Å
β = 95.126 (1)°
*V* = 1445.23 (4) Å^3^
*Z* = 8
Mo *K*α radiation
μ = 0.09 mm^−1^
*T* = 100 K
0.40 × 0.20 × 0.05 mm

#### Data collection

Bruker SMART APEXII CCD diffractometer
Absorption correction: multi-scan (*SADABS*; Bruker, 2009 ▶) *T*_min_ = 0.964, *T*_max_ = 0.995
14322 measured reflections
4231 independent reflections
3120 reflections with *I* > 2σ(*I*)
*R*_int_ = 0.040

#### Refinement

*R*[*F* ^2^ > 2σ(*F* ^2^)] = 0.049
*wR*(*F* ^2^) = 0.114
*S* = 1.00
4231 reflections
205 parameters
H atoms treated by a mixture of independent and constrained refinement
Δρ_max_ = 0.28 e Å^−3^
Δρ_min_ = −0.30 e Å^−3^

### 

Data collection: *APEX2* (Bruker, 2009 ▶); cell refinement: *SAINT* (Bruker, 2009 ▶); data reduction: *SAINT*; program(s) used to solve structure: *SHELXTL* (Sheldrick, 2008 ▶); program(s) used to refine structure: *SHELXTL*; molecular graphics: *SHELXTL*; software used to prepare material for publication: *SHELXTL* and *PLATON* (Spek, 2009 ▶).

## Supplementary Material

Crystal structure: contains datablock(s) global, I. DOI: 10.1107/S1600536812034599/hb6915sup1.cif

Structure factors: contains datablock(s) I. DOI: 10.1107/S1600536812034599/hb6915Isup2.hkl

Supplementary material file. DOI: 10.1107/S1600536812034599/hb6915Isup3.cml

Additional supplementary materials:  crystallographic information; 3D view; checkCIF report

## Figures and Tables

**Table 1:** Hydrogen-bond geometry (Å, °)

*D*—H⋯*A*	*D*—H	H⋯*A*	*D*⋯*A*	*D*—H⋯*A*
N2*A*—H1*N*2⋯O1*B*	0.875 (17)	2.048 (17)	2.9088 (14)	167.7 (17)
N3*A*—H1*N*3⋯N1*B* ^i^	0.858 (18)	2.614 (18)	3.3214 (16)	140.5 (16)
N3*A*—H2*N*3⋯O1*A* ^ii^	0.926 (19)	1.949 (19)	2.8749 (16)	178.7 (15)
N2*B*—H2*N*2⋯O1*A*	0.919 (17)	2.065 (17)	2.9663 (14)	166.6 (16)
N3*B*—H3*N*3⋯O1*B* ^iii^	0.889 (19)	1.980 (19)	2.8682 (16)	175.9 (18)
N3*B*—H4*N*3⋯N1*A* ^iv^	0.858 (17)	2.515 (17)	3.1771 (16)	134.7 (15)
C1*A*—H1*AB*⋯O1*B* ^v^	0.99	2.52	3.3923 (18)	146

Symmetry codes: (i) 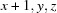; (ii) 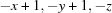; (iii) 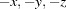; (iv) 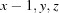; (v) 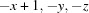.

**Fig. 1.**
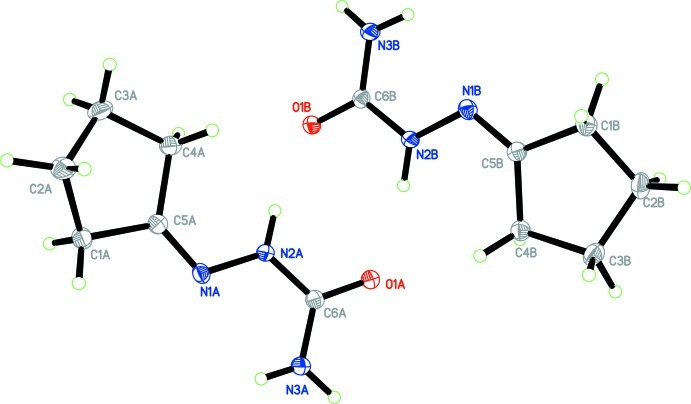
The molecular structure of the title compound, showing 50% probability displacement ellipsoids.

**Fig. 2.**
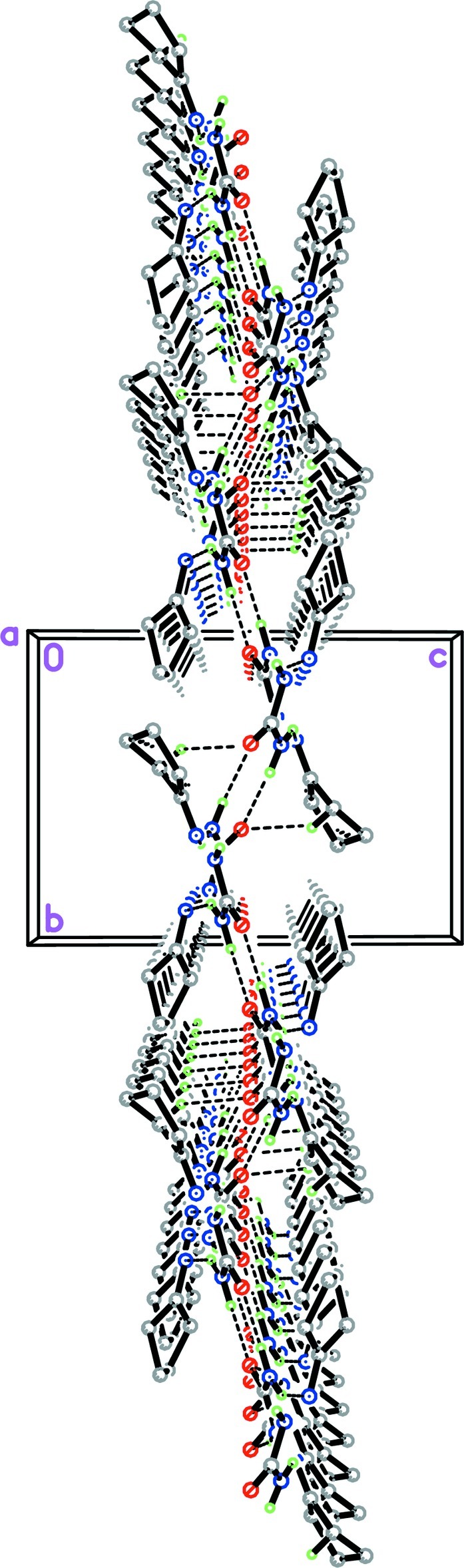
The crystal packing of the title compound, viewed along the a axis. H atoms not involved in the intermolecular interactions (dashed lines) have been omitted for clarity.

## supplementary crystallographic information

### Comment

Various semicarbazones, have been known to possess biological activities against many of the most common species of bacteria (Dogan *et al.*, 1999). Semicarbazones are of much interest due to their wide spectrum of antibacterial activities (Pandeya & Dimmock, 1993). Recently some workers reviewed the bioactivity of semicarbazones and they have exhibited anticonvulsant (Pandeya *et al.*, 1998; Yogeeswari *et al.*, 2004) and antitubercular (Sriram *et al.*, 2004) properties. Our previous report highlights the synthesis and crystal structures of the semicarbozones (Fun *et al.*, 2011). In continuation of our studies in this area, we now report the synthesis and structure of the title compound.

The asymmetric unit of the title compound, Fig. 1, consists of two crystallographically independent molecules. The cyclopentane (C1–C5) rings adopt an envelope conformation. The semicarbazone groups (O1/N1–N3/C6) are essentially planar with maximum deviation of 0.0311 (12) Å at atom N2A and 0.0285 (12) Å at atom N2B. Bond lengths and angles are comparable with the related structures (Fun *et al.* 2009**a*,b*).

In the crystal, Fig. 2, N2A—H1N2···O1B, N3A—H1N3···N1B, N3A—H2N3···O1A, N2B—H2N2···O1A, N3B—H3N3···O1B, N3B—H4N3···N1A and C1A—H1AB···O1B hydrogen bonds (Table 1), link the molecules to form planes parallel to the *ab* plane.

### Experimental

Semicarbazide hydrochloride (0.66 g, 0.0059 mol) and freshly recrystallized sodium acetate (0.58 g, 0.007 mol) were dissolved in water (10 ml) following a literature procedure (Furniss *et al.*, 1978). The reaction mixture was stirred at room temperature for 10 minutes. To this, cyclopentanone (0.5 g, 0.0059 mol) was added and shaken well. A little alcohol was added to dissolve the turbidity. It was shaken for 10 more minutes and allowed to stand. The semicarbazone crystallized on standing for 6 h. The separated crystals were filtered, washed with cold water and recrystallized from ethanol as colourless plates. *M.p.* 495–498 K.

### Refinement

N– bound H atoms were located from the difference Fourier map and were refined freely [N–H = 0.858 (18) to 0.926 (19) Å]. The remaining H atoms were located geometrically and were refined using a riding model with *U*_iso_(H) = 1.2 *U*_eq_(C) [C–H = 0.99 Å]. In the final refinement, one outliner was omitted, 6 11 8.

### Crystal data

**Table:**

C_6_H_11_N_3_O	*F*(000) = 608
*M**_r_* = 141.18	*D*_x_ = 1.298 Mg m^−^^3^
Monoclinic, *P*2_1_/*c*	Mo *K*α radiation, λ = 0.71073 Å
Hall symbol: -P 2ybc	Cell parameters from 3109 reflections
*a* = 8.9507 (1) Å	θ = 2.7–29.3°
*b* = 10.7929 (2) Å	µ = 0.09 mm^−^^1^
*c* = 15.0204 (2) Å	*T* = 100 K
β = 95.126 (1)°	Plate, colourless
*V* = 1445.23 (4) Å^3^	0.40 × 0.20 × 0.05 mm
*Z* = 8	

### Data collection

**Table:**

Bruker SMART APEXII CCD diffractometer	4231 independent reflections
Radiation source: fine-focus sealed tube	3120 reflections with *I* > 2σ(*I*)
Graphite monochromator	*R*_int_ = 0.040
φ and ω scans	θ_max_ = 30.1°, θ_min_ = 2.3°
Absorption correction: multi-scan (*SADABS*; Bruker, 2009)	*h* = −12→12
*T*_min_ = 0.964, *T*_max_ = 0.995	*k* = −12→15
14322 measured reflections	*l* = −18→21

### Refinement

**Table:**

Refinement on *F*^2^	Primary atom site location: structure-invariant direct methods
Least-squares matrix: full	Secondary atom site location: difference Fourier map
*R*[*F*^2^ > 2σ(*F*^2^)] = 0.049	Hydrogen site location: inferred from neighbouring sites
*wR*(*F*^2^) = 0.114	H atoms treated by a mixture of independent and constrained refinement
*S* = 1.00	*w* = 1/[σ^2^(*F*_o_^2^) + (0.0422*P*)^2^ + 0.6439*P*] where *P* = (*F*_o_^2^ + 2*F*_c_^2^)/3
4231 reflections	(Δ/σ)_max_ < 0.001
205 parameters	Δρ_max_ = 0.28 e Å^−^^3^
0 restraints	Δρ_min_ = −0.30 e Å^−^^3^

### Special details

**Table:**

Experimental. The crystal was placed in the cold stream of an Oxford Cryosystems Cobra open-flow nitrogen cryostat (Cosier & Glazer, 1986) operating at 100.0 (1) K.
Geometry. All e.s.d.'s (except the e.s.d. in the dihedral angle between two l.s. planes) are estimated using the full covariance matrix. The cell e.s.d.'s are taken into account individually in the estimation of e.s.d.'s in distances, angles and torsion angles; correlations between e.s.d.'s in cell parameters are only used when they are defined by crystal symmetry. An approximate (isotropic) treatment of cell e.s.d.'s is used for estimating e.s.d.'s involving l.s. planes.
Refinement. Refinement of *F*^2^ against ALL reflections. The weighted *R*-factor *wR* and goodness of fit *S* are based on *F*^2^, conventional *R*-factors *R* are based on *F*, with *F* set to zero for negative *F*^2^. The threshold expression of *F*^2^ > σ(*F*^2^) is used only for calculating *R*-factors(gt) *etc*. and is not relevant to the choice of reflections for refinement. *R*-factors based on *F*^2^ are statistically about twice as large as those based on *F*, and *R*- factors based on ALL data will be even larger.

### Fractional atomic coordinates and isotropic or equivalent isotropic displacement parameters (Å^2^)

**Table:**

	*x*	*y*	*z*	*U*_iso_*/*U*_eq_	
O1B	0.14864 (10)	0.11961 (9)	0.00969 (7)	0.0163 (2)	
N1B	−0.03324 (12)	0.35060 (10)	0.12404 (8)	0.0142 (2)	
N2B	0.07174 (12)	0.29357 (11)	0.07505 (8)	0.0148 (2)	
N3B	−0.07584 (13)	0.11717 (12)	0.06934 (9)	0.0180 (3)	
C1B	−0.11403 (15)	0.53548 (13)	0.19876 (10)	0.0179 (3)	
H1BA	−0.0892	0.5196	0.2633	0.021*	
H1BB	−0.2200	0.5125	0.1824	0.021*	
C2B	−0.08570 (15)	0.67107 (13)	0.17588 (10)	0.0196 (3)	
H2BA	−0.1079	0.7264	0.2256	0.024*	
H2BB	−0.1479	0.6964	0.1211	0.024*	
C3B	0.08159 (15)	0.67431 (13)	0.16132 (10)	0.0187 (3)	
H3BA	0.1436	0.6815	0.2190	0.022*	
H3BB	0.1043	0.7450	0.1227	0.022*	
C4B	0.11103 (14)	0.55001 (12)	0.11528 (9)	0.0154 (3)	
H4BA	0.1036	0.5597	0.0495	0.018*	
H4BB	0.2117	0.5175	0.1357	0.018*	
C5B	−0.01062 (14)	0.46502 (12)	0.14338 (9)	0.0140 (3)	
C6B	0.05040 (14)	0.17321 (12)	0.04950 (9)	0.0132 (3)	
O1A	0.35640 (10)	0.37841 (9)	0.00812 (7)	0.0169 (2)	
N1A	0.57800 (12)	0.13301 (11)	0.09989 (8)	0.0145 (2)	
N2A	0.45488 (12)	0.19836 (11)	0.06156 (8)	0.0153 (3)	
N3A	0.60763 (13)	0.36934 (12)	0.04762 (9)	0.0194 (3)	
C1A	0.67282 (15)	−0.05543 (13)	0.17484 (10)	0.0173 (3)	
H1AA	0.7522	−0.0047	0.2072	0.021*	
H1AB	0.7184	−0.1093	0.1313	0.021*	
C2A	0.58742 (15)	−0.13169 (14)	0.23954 (10)	0.0202 (3)	
H2AA	0.5718	−0.0837	0.2941	0.024*	
H2AB	0.6417	−0.2091	0.2569	0.024*	
C3A	0.43811 (16)	−0.15927 (14)	0.18545 (10)	0.0208 (3)	
H3AA	0.3591	−0.1788	0.2254	0.025*	
H3AB	0.4482	−0.2299	0.1444	0.025*	
C4A	0.40070 (15)	−0.03906 (13)	0.13277 (10)	0.0176 (3)	
H4AA	0.3551	−0.0577	0.0718	0.021*	
H4AB	0.3307	0.0133	0.1637	0.021*	
C5A	0.55099 (14)	0.02476 (12)	0.12953 (9)	0.0137 (3)	
C6A	0.47038 (14)	0.31899 (12)	0.03773 (9)	0.0138 (3)	
H1N2	0.3656 (19)	0.1655 (16)	0.0516 (12)	0.028 (5)*	
H1N3	0.682 (2)	0.3267 (17)	0.0710 (12)	0.030 (5)*	
H2N3	0.6182 (19)	0.4510 (18)	0.0303 (12)	0.028 (5)*	
H2N2	0.1582 (19)	0.3309 (16)	0.0600 (12)	0.027 (5)*	
H3N3	−0.0968 (19)	0.0420 (18)	0.0474 (12)	0.028 (5)*	
H4N3	−0.1441 (19)	0.1587 (16)	0.0927 (12)	0.026 (5)*	

### Atomic displacement parameters (Å^2^)

**Table:**

	*U*^11^	*U*^22^	*U*^33^	*U*^12^	*U*^13^	*U*^23^
O1B	0.0139 (4)	0.0136 (5)	0.0217 (5)	0.0001 (3)	0.0040 (4)	−0.0029 (4)
N1B	0.0147 (5)	0.0156 (6)	0.0128 (5)	0.0013 (4)	0.0032 (4)	−0.0006 (4)
N2B	0.0128 (5)	0.0126 (6)	0.0197 (6)	−0.0009 (4)	0.0059 (4)	−0.0018 (5)
N3B	0.0165 (5)	0.0131 (6)	0.0255 (7)	−0.0022 (5)	0.0082 (5)	−0.0039 (5)
C1B	0.0174 (6)	0.0177 (7)	0.0189 (7)	−0.0005 (5)	0.0038 (5)	−0.0045 (6)
C2B	0.0204 (6)	0.0168 (7)	0.0214 (7)	0.0043 (5)	0.0000 (5)	−0.0050 (6)
C3B	0.0205 (6)	0.0138 (7)	0.0216 (7)	−0.0006 (5)	−0.0001 (6)	−0.0026 (6)
C4B	0.0153 (6)	0.0150 (6)	0.0158 (7)	0.0005 (5)	0.0015 (5)	−0.0005 (5)
C5B	0.0141 (6)	0.0143 (6)	0.0136 (6)	0.0001 (5)	0.0002 (5)	0.0007 (5)
C6B	0.0138 (6)	0.0127 (6)	0.0128 (6)	0.0006 (5)	−0.0006 (5)	0.0009 (5)
O1A	0.0136 (4)	0.0144 (5)	0.0226 (5)	0.0005 (4)	0.0006 (4)	0.0020 (4)
N1A	0.0127 (5)	0.0149 (6)	0.0161 (6)	0.0025 (4)	0.0026 (4)	0.0001 (5)
N2A	0.0113 (5)	0.0129 (6)	0.0216 (6)	−0.0004 (4)	0.0007 (4)	0.0031 (5)
N3A	0.0132 (5)	0.0153 (6)	0.0293 (7)	−0.0007 (4)	0.0001 (5)	0.0057 (5)
C1A	0.0156 (6)	0.0190 (7)	0.0175 (7)	0.0021 (5)	0.0034 (5)	0.0042 (6)
C2A	0.0193 (7)	0.0233 (8)	0.0181 (7)	−0.0014 (6)	0.0032 (5)	0.0063 (6)
C3A	0.0213 (7)	0.0185 (7)	0.0225 (7)	−0.0054 (5)	0.0013 (6)	0.0064 (6)
C4A	0.0159 (6)	0.0174 (7)	0.0195 (7)	−0.0022 (5)	0.0019 (5)	0.0022 (6)
C5A	0.0149 (6)	0.0149 (7)	0.0118 (6)	0.0005 (5)	0.0036 (5)	−0.0012 (5)
C6A	0.0145 (6)	0.0143 (7)	0.0131 (6)	0.0001 (5)	0.0039 (5)	−0.0019 (5)

### Geometric parameters (Å, º)

**Table:**

O1B—C6B	1.2488 (16)	O1A—C6A	1.2526 (15)
N1B—C5B	1.2806 (17)	N1A—C5A	1.2811 (18)
N1B—N2B	1.3882 (16)	N1A—N2A	1.3892 (14)
N2B—C6B	1.3631 (17)	N2A—C6A	1.3605 (17)
N2B—H2N2	0.919 (17)	N2A—H1N2	0.875 (17)
N3B—C6B	1.3385 (17)	N3A—C6A	1.3394 (17)
N3B—H3N3	0.889 (19)	N3A—H1N3	0.860 (18)
N3B—H4N3	0.858 (18)	N3A—H2N3	0.926 (19)
C1B—C5B	1.5049 (19)	C1A—C5A	1.5062 (17)
C1B—C2B	1.530 (2)	C1A—C2A	1.529 (2)
C1B—H1BA	0.9900	C1A—H1AA	0.9900
C1B—H1BB	0.9900	C1A—H1AB	0.9900
C2B—C3B	1.533 (2)	C2A—C3A	1.5300 (18)
C2B—H2BA	0.9900	C2A—H2AA	0.9900
C2B—H2BB	0.9900	C2A—H2AB	0.9900
C3B—C4B	1.5427 (19)	C3A—C4A	1.541 (2)
C3B—H3BA	0.9900	C3A—H3AA	0.9900
C3B—H3BB	0.9900	C3A—H3AB	0.9900
C4B—C5B	1.5124 (19)	C4A—C5A	1.5159 (18)
C4B—H4BA	0.9900	C4A—H4AA	0.9900
C4B—H4BB	0.9900	C4A—H4AB	0.9900
			
C5B—N1B—N2B	116.56 (11)	C5A—N1A—N2A	116.09 (11)
C6B—N2B—N1B	119.17 (11)	C6A—N2A—N1A	119.94 (11)
C6B—N2B—H2N2	116.7 (11)	C6A—N2A—H1N2	117.2 (12)
N1B—N2B—H2N2	124.1 (11)	N1A—N2A—H1N2	122.9 (12)
C6B—N3B—H3N3	118.9 (12)	C6A—N3A—H1N3	119.9 (12)
C6B—N3B—H4N3	120.1 (12)	C6A—N3A—H2N3	118.1 (10)
H3N3—N3B—H4N3	119.6 (16)	H1N3—N3A—H2N3	121.9 (16)
C5B—C1B—C2B	103.70 (12)	C5A—C1A—C2A	102.33 (11)
C5B—C1B—H1BA	111.0	C5A—C1A—H1AA	111.3
C2B—C1B—H1BA	111.0	C2A—C1A—H1AA	111.3
C5B—C1B—H1BB	111.0	C5A—C1A—H1AB	111.3
C2B—C1B—H1BB	111.0	C2A—C1A—H1AB	111.3
H1BA—C1B—H1BB	109.0	H1AA—C1A—H1AB	109.2
C1B—C2B—C3B	103.74 (11)	C1A—C2A—C3A	103.28 (11)
C1B—C2B—H2BA	111.0	C1A—C2A—H2AA	111.1
C3B—C2B—H2BA	111.0	C3A—C2A—H2AA	111.1
C1B—C2B—H2BB	111.0	C1A—C2A—H2AB	111.1
C3B—C2B—H2BB	111.0	C3A—C2A—H2AB	111.1
H2BA—C2B—H2BB	109.0	H2AA—C2A—H2AB	109.1
C2B—C3B—C4B	104.61 (11)	C2A—C3A—C4A	104.38 (11)
C2B—C3B—H3BA	110.8	C2A—C3A—H3AA	110.9
C4B—C3B—H3BA	110.8	C4A—C3A—H3AA	110.9
C2B—C3B—H3BB	110.8	C2A—C3A—H3AB	110.9
C4B—C3B—H3BB	110.8	C4A—C3A—H3AB	110.9
H3BA—C3B—H3BB	108.9	H3AA—C3A—H3AB	108.9
C5B—C4B—C3B	104.26 (11)	C5A—C4A—C3A	104.26 (10)
C5B—C4B—H4BA	110.9	C5A—C4A—H4AA	110.9
C3B—C4B—H4BA	110.9	C3A—C4A—H4AA	110.9
C5B—C4B—H4BB	110.9	C5A—C4A—H4AB	110.9
C3B—C4B—H4BB	110.9	C3A—C4A—H4AB	110.9
H4BA—C4B—H4BB	108.9	H4AA—C4A—H4AB	108.9
N1B—C5B—C1B	121.34 (12)	N1A—C5A—C1A	121.98 (11)
N1B—C5B—C4B	128.67 (13)	N1A—C5A—C4A	128.40 (12)
C1B—C5B—C4B	109.97 (11)	C1A—C5A—C4A	109.46 (11)
O1B—C6B—N3B	122.81 (12)	O1A—C6A—N3A	122.79 (13)
O1B—C6B—N2B	119.30 (12)	O1A—C6A—N2A	118.98 (11)
N3B—C6B—N2B	117.89 (12)	N3A—C6A—N2A	118.24 (12)
			
C5B—N1B—N2B—C6B	−177.45 (11)	C5A—N1A—N2A—C6A	172.46 (13)
C5B—C1B—C2B—C3B	−34.21 (13)	C5A—C1A—C2A—C3A	38.89 (14)
C1B—C2B—C3B—C4B	36.78 (14)	C1A—C2A—C3A—C4A	−38.43 (15)
C2B—C3B—C4B—C5B	−24.62 (14)	C2A—C3A—C4A—C5A	22.39 (15)
N2B—N1B—C5B—C1B	−177.98 (11)	N2A—N1A—C5A—C1A	−178.43 (12)
N2B—N1B—C5B—C4B	4.09 (19)	N2A—N1A—C5A—C4A	−3.6 (2)
C2B—C1B—C5B—N1B	−158.97 (12)	C2A—C1A—C5A—N1A	150.21 (13)
C2B—C1B—C5B—C4B	19.31 (13)	C2A—C1A—C5A—C4A	−25.50 (15)
C3B—C4B—C5B—N1B	−178.62 (13)	C3A—C4A—C5A—N1A	−173.30 (14)
C3B—C4B—C5B—C1B	3.26 (13)	C3A—C4A—C5A—C1A	2.06 (15)
N1B—N2B—C6B—O1B	−176.11 (11)	N1A—N2A—C6A—O1A	−175.88 (12)
N1B—N2B—C6B—N3B	3.25 (18)	N1A—N2A—C6A—N3A	3.7 (2)

### Hydrogen-bond geometry (Å, º)

**Table:**

*D*—H···*A*	*D*—H	H···*A*	*D*···*A*	*D*—H···*A*
N2*A*—H1*N*2···O1*B*	0.875 (17)	2.048 (17)	2.9088 (14)	167.7 (17)
N3*A*—H1*N*3···N1*B*^i^	0.858 (18)	2.614 (18)	3.3214 (16)	140.5 (16)
N3*A*—H2*N*3···O1*A*^ii^	0.926 (19)	1.949 (19)	2.8749 (16)	178.7 (15)
N2*B*—H2*N*2···O1*A*	0.919 (17)	2.065 (17)	2.9663 (14)	166.6 (16)
N3*B*—H3*N*3···O1*B*^iii^	0.889 (19)	1.980 (19)	2.8682 (16)	175.9 (18)
N3*B*—H4*N*3···N1*A*^iv^	0.858 (17)	2.515 (17)	3.1771 (16)	134.7 (15)
C1*A*—H1*AB*···O1*B*^v^	0.99	2.52	3.3923 (18)	146

Symmetry codes: (i) *x*+1, *y*, *z*; (ii) −*x*+1, −*y*+1, −*z*; (iii) −*x*, −*y*, −*z*; (iv) *x*−1, *y*, *z*; (v) −*x*+1, −*y*, −*z*.
